# Tat-Biliverdin Reductase A Exerts a Protective Role in Oxidative Stress-Induced Hippocampal Neuronal Cell Damage by Regulating the Apoptosis and MAPK Signaling

**DOI:** 10.3390/ijms21082672

**Published:** 2020-04-11

**Authors:** Sang Jin Kim, Min Jea Shin, Dae Won Kim, Hyeon Ji Yeo, Eun Ji Yeo, Yeon Joo Choi, Eun Jeong Sohn, Kyu Hyung Han, Jinseu Park, Keun Wook Lee, Jong Kook Park, Yong-Jun Cho, Duk-Soo Kim, Won Sik Eum, Soo Young Choi

**Affiliations:** 1 Department of Biomedical Science and Research, Institute of Bioscience and Biotechnology, Hallym University, Chuncheon 24252, Korea; oksangjin@naver.com (S.J.K.); wehome3@hallym.ac.kr (M.J.S.); hj0428@hallym.ac.kr (H.J.Y.); ej428@hallym.ac.kr (E.J.Y); cyj0036@hallym.ac.kr (Y.J.C.); ejsohn84@hallym.ac.kr (E.J.S.); khhan@hallym.ac.kr (K.H.H.); jinpark@hallym.ac.kr (J.P.); keunwook@hallym.ac.kr (K.W.L); jkp555@hallym.ac.kr (J.K.P.); 2 Department of Biochemistry and Molecular Biology, Research Institute of Oral Sciences, College of Dentistry, Gangneung-Wonju National University, Gangneung 25457, Korea; kimdw@gwnu.ac.kr; 3 Department of Neurosurgery, Hallym University Medical Center, Chuncheon 24253, Korea; nssur771@hallym.or.kr; 4 Department of Anatomy, College of Medicine, Soonchunhyang University, Cheonan-Si 31538, Korea; dskim@sch.ac.kr

**Keywords:** Tat-BLVRA, oxidative stress, MAPK, ischemic injury, protein therapy

## Abstract

Reactive oxygen species (ROS) is major risk factor in neuronal diseases including ischemia. Although biliverdin reductase A (BLVRA) plays a pivotal role in cell survival via its antioxidant function, its role in hippocampal neuronal (HT-22) cells and animal ischemic injury is not clearly understood yet. In this study, the effects of transducible fusion protein Tat-BLVRA on H_2_O_2_-induced HT-22 cell death and in an animal ischemia model were investigated. Transduced Tat-BLVRA markedly inhibited cell death, DNA fragmentation, and generation of ROS. Transduced Tat-BLVRA inhibited the apoptosis and mitogen activated protein kinase (MAPK) signaling pathway and it passed through the blood-brain barrier (BBB) and significantly prevented hippocampal cell death in an ischemic model. These results suggest that Tat-BLVRA provides a possibility as a therapeutic molecule for ischemia.

## 1. Introduction

Biliverdin reductase is known as an evolutionarily conserved soluble protein which is found in various species, the biological function of biliverdin reductase is to convert biliverdin to bilirubin in the heme metabolism pathway [1]. Biliverdin reductase has two isozymes, biliverdin reductase A (BLVRA) and biliverdin reductase B (BLVRB), and BLVRA mRNA was abundantly expressed in various tissues [2,3]. Other studies have demonstrated that biliverdin reductase and enzyme product bilirubin have antioxidant functions by reducing the reactive oxygen species (ROS) [4,5].

Biliverdin reductase and bilirubin are involved in various diseases, including brain damage and protection against oxidative stress-induced neuronal injury [6,7,8,9]. BLVRA has an antioxidant function on ROS via production of bilirubin. Bilirubin, as a powerful antioxidant, protects against H_2_O_2_-induced cultured neuronal cells [4,10]. Oxidative stress-induced impairment of BLVRA increased accumulation of amyloid beta (Aβ) and tumor necrosis factor-alpha (TNF-α), that greatly contribute to the onset of brain insulin resistance along the progression of Alzheimer’s disease pathology [11]. Similarly, reduced BLVRA levels increased oxidative stress and Tau phosphorylation in young triple transgenic AD (3xTg-AD)mice, suggesting loss of BLVRA impaired neuroprotection in response to oxidative stress in Alzheimer’s disease (AD) [9]. In experimental autoimmune encephalomyelitis, biliverdin reductase more efficiently reduced clinical and pathological signs than treatments with other antioxidant enzymes in SH-SY5Y cells and in a Rat model [11]. In addition, biliverdin reductase and bilirubin are involved in the regulation of MAPK, phosphatidylinositol 3-hydroxy kinase/protein kinase B (PI3K/Akt), and protein kinase C delta (PKCδ) signaling pathways and various gene expressions (growth regulators, differentiation factors, and transcription factors) related to cell survival, suggesting that biliverdin reductase may be a potential therapeutic agent for various diseases [9,10,11,12,13,14,15].

Oxidative stress induces cellular ROS generation, excessive elevation of neuronal cell death by modification of cellular macromolecules, including DNA and proteins [16,17,18]. Excessive elevation of ROS in neuronal cells is highly associated with apoptosis and causes neurodegenerative diseases, including ischemia [16,17,18,19,20,21,22,23,24].

Protein transduction domains (PTDs) are well known to deliver proteins into cells. PTDs have been used to apply the development protein therapy for various diseases [22,25,26,27,28,29,30,31,32,33,34,35,36]. Here, we examined the effect of Tat-BLVRA against oxidative stress-induced hippocampal neuronal cell death and in an insult animal model of ischemia. 

## 2. Results

### 2.1. Purification and Transduction of Tat-BLVRA into HT-22 Cells

**Figure 1:**
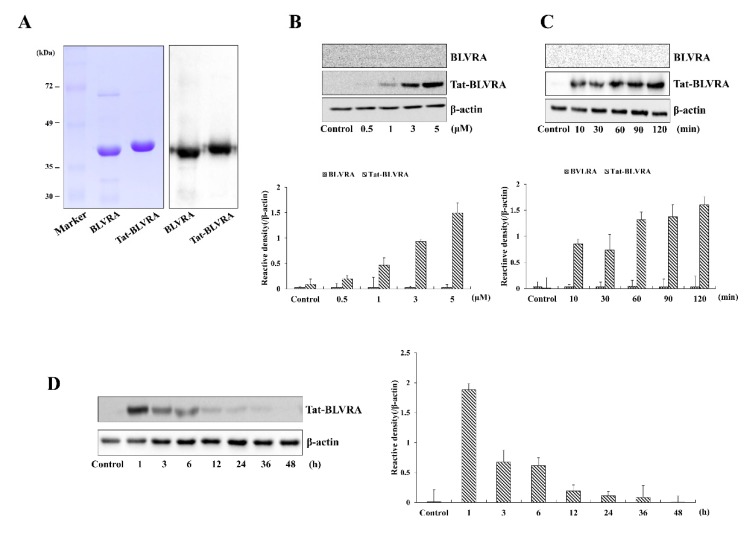
Purification and transduction of Tat-BLVRA protein. Purification of Tat-BLVRA and control BLVRA proteins. Purified proteins were analyzed by sodium dodecyl sulfate-polyacrylamide-gel electrophoresis (SDS-PAGE) and subjected to Western blot analysis with anti-Histidine antibody (**A**). Transduction of Tat-BLVRA proteins into HT-22 cells. Tat-BLVRA or control BLVRA (0.5–5 µM) proteins were added to the culture medium for 2 h (**B**). Tat-BLVRA or control BLVRA (5 µM) proteins were added to the culture medium for 10–120 min (**C**). Intracellular stability of transduced Tat-BLVRA (**D**). Cells were exposed to Tat-BLVRA (5 µM) protein for 2 h and over various time periods. Then, the levels of Tat-BLVRA protein were measured by Western blotting and band intensity was assessed by densitometer. The bars in the figures represent the mean ± standard error of the mean (SEM) obtained from 3 independent experiments.

Figure 1A shows the purified Tat- and control-BLVRA. Hippocampal neuronal (HT-22) cells were treated with Tat-BLVRA or control BLVRA (0.5–5 µM) for 2 h or with Tat-BLVRA or control BLVRA (5 µM) for various times (10–120 min). Then, transduced Tat-BLVRA and control BLVRA levels were determined (Figure 1B,C). Transduced Tat-BLVRA levels increased in concentration- and time-dependent manners, whereas control BLVRA showed no evidence of transduction. Figure 1D shows that Tat-BLVRA persisted for 6 h in HT-22 cells.

### 2.2. Effect of Tat-BLVRA against H_2_O_2_-Induced Cell Death

**Figure 2:**
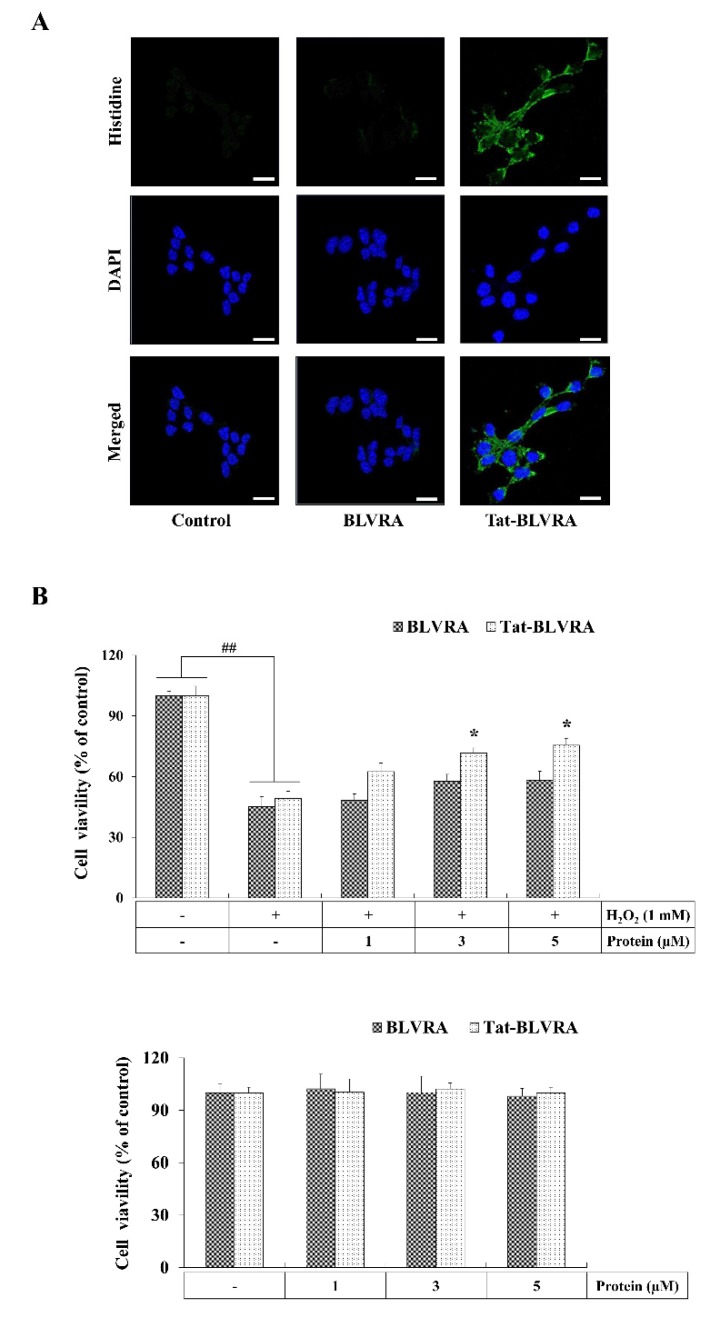
Effect of transduced Tat-BLVRA protein against H_2_O_2_-induced cell death. Cellular distribution of transduced Tat-BLVRA protein in HT-22 cells (**A**). Cells were exposed to Tat-BLVRA and control BLVRA protein (5 µM) for 2 h and the distribution of the transduced Tat-BLVRA protein was observed by confocal microscopy. Scale bar = 50 μm. Cell viabilities were assessed by 3-(4,5-dimethylthiazol-2-yl)-2,5-diphenyl terazolium bromide (MTT) assay (**B**). HT-22 cells were treated with Tat-BLVRA and control BLVRA protein (1–5 µM) for 2 h, after which cells were incubated with or without 1 mM hydrogen peroxide for 2.5 h. The absorbance was measured at 570 nm using an enzyme-linked immunosorbent assay (ELISA) microplate reader and the cell viability was defined as the % of untreated control cells. The bars in the figures represent the mean ± SEM obtained from 3 independent experiments. * *p* < 0.05 compared to cells treated only with H_2_O_2_. ^##^ *p* < 0.01 compared to the untreated control cells.

We examined whether transduced Tat-BLVRA inhibits H_2_O_2_-induced HT-22 cell death. Cells were exposed to Tat-BLVRA and control proteins (5 µM) for 2 h. Using 4’,6-diamidino-2-phenylindole (DAPI) and antibodies against His-tagged protein, transduced Tat-BLVRA was evident in both the nucleus and cytosol (Figure 2A), whereas control BLVRA did not transduce into cells.

The viability of cells which were treated with H_2_O_2_ (1 mM) for 2.5 h was determined after pretreatment of Tat-BLVRA (1–5 μM). Transduced Tat-BLVRA increased cell survival in a concentration-dependent manner up to 75% in the cells. In contrast, treatment with control BLVRA did not have any protective effect. Transduced Tat-BLVRA did not exert a toxic effect in the cells without H_2_O_2_ (Figure 2B).

### 2.3. Protective Effect of Tat-BLVRA against H_2_O_2_-Induced Cytotoxicity

**Figure 3:**
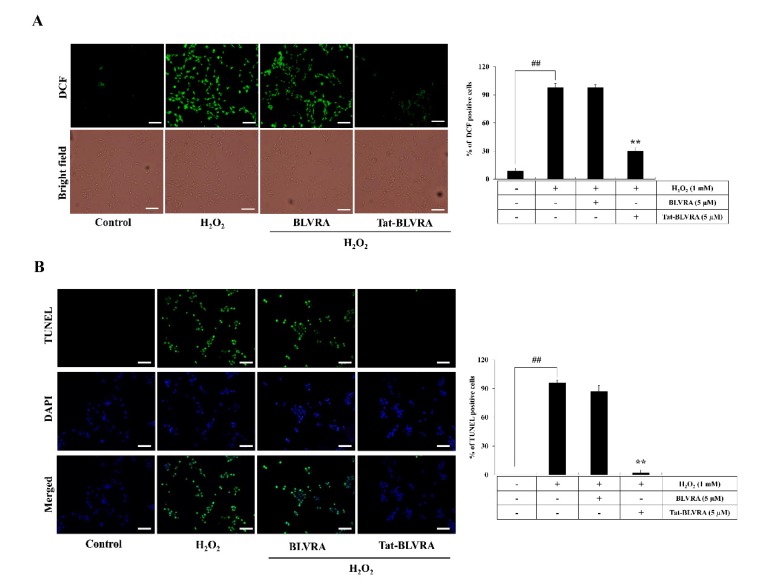
Effect of Tat-BLVRA protein against H_2_O_2_-induced cellular toxicity. Tat-BLVRA or control BLVRA proteins (5 µM) were added to the culture medium and exposed to H_2_O_2_. Reactive oxygen species (ROS) levels were measured using 2′,7′-dichlorodihydrofluorescein diacetate (DCF-DA) staining (**A**). DNA fragmentation was detected by terminal deoxynucleotidyl transferase mediated dUTP nick end labeling (TUNEL) staining and quantitative evaluation of TUNEL-positive cells was confirmed by cell counting under a phase-contrast microscope (×200 magnification) (**B**). The fluorescence intensity was measured by an ELISA plate reader. The bars in the figures represent the mean ± SEM obtained from 3 independent experiments. ** *p* < 0.01 compared to cells treated only with H_2_O_2_. ^##^ *p* < 0.01 compared to the untreated control cells. Scale bar = 50 μm.

Further, we confirmed ROS production and DNA damage. In Figure 3A, B, strong fluorescence signals appeared in the H_2_O_2_-only treated cells, whereas Tat-BLVRA significantly reduced fluorescence compared to those of control BLVRA protein or H_2_O_2_-only treated cells.

### 2.4. Effects of Tat-BLVRA on H_2_O_2_-Induced Activation of MAPKs and Apoptosis

**Figure 4:**
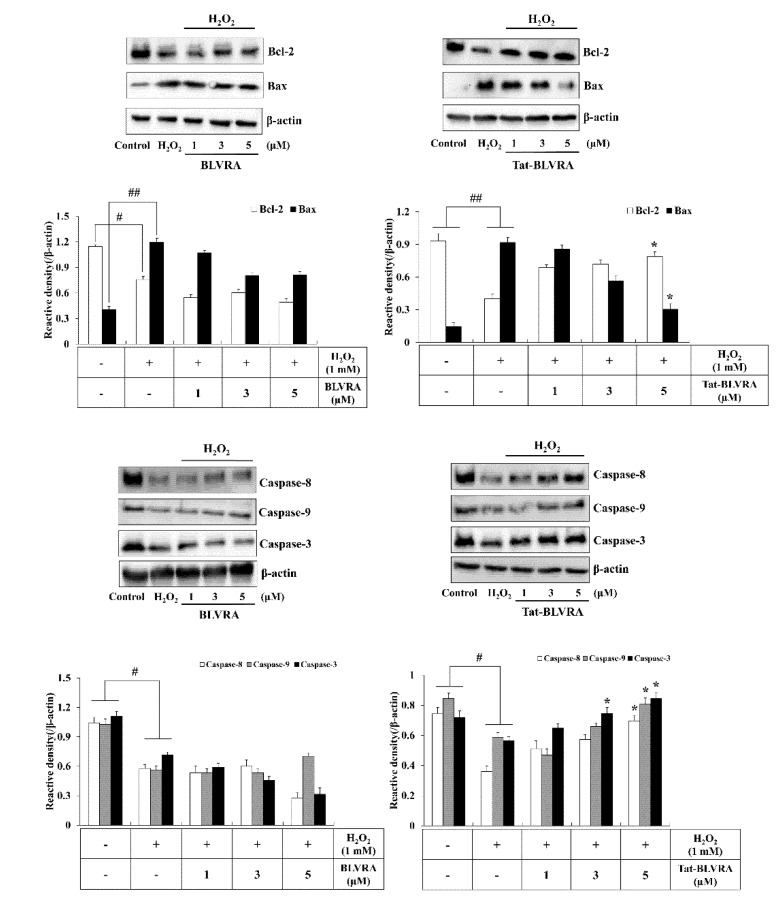
Effect of Tat-BLVRA protein on the expression of Bcl-2, Bax, and caspase cascades in HT-22 cells. The cells were treated with Tat-BLVRA protein and then exposed to H_2_O_2_. The expression of Bcl-2 and Bax as well as caspase cascade levels were measured by Western blotting and band intensity was measured by a densitometer. The bars in the figures represent the mean ± SEM obtained from 3 independent experiments. * *p* < 0.05 compared to cells treated only with H_2_O_2_. ^#^ *p* < 0.05 and ^##^ *p* < 0.01 compared to the untreated control cells.

Since changes in anti- or pro-apoptosis protein expression levels induced by oxidative stress are related to cell survival [37,38], we investigated the changes of those proteins by Tat-BLVRA H_2_O_2_-exposed HT-22 cells. Tat-BLVRA increased Bcl-2 expression levels, whereas Bax expression levels were decreased. Also, Tat-BLVRA increased caspase-8, -9, and -3 expression levels in a dose-dependent manner in HT-22 cells exposed to H_2_O_2_. However, control BLVRA did not change anti- or pro-apoptosis protein expression levels (Figure 4).

**Figure 5:**
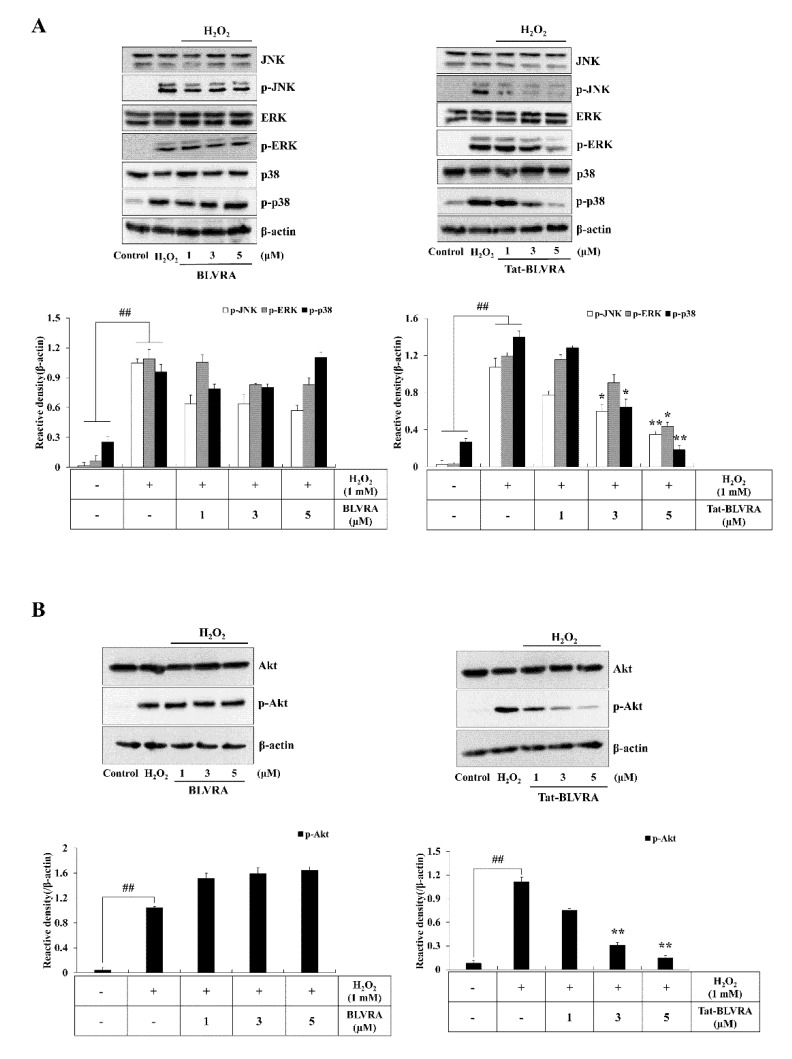
Effect of Tat-BLVRA protein on the activation of MAPK (**A**) and protein kinase B (Akt) (**B**) in HT-22 cells. The cells were treated with Tat-BLVRA protein and then exposed to H_2_O_2_. The activation of MAPK and Akt levels were measured by Western blotting and band intensity was measured by a densitometer. The bars in the figures represent the mean ± SEM obtained from 3 independent experiments. * *p* < 0.05 and ** *p* < 0.01 compared to cells treated only with H_2_O_2_. ^##^ *p* < 0.01 compared to the untreated control cells.

It has been reported that cell death is caused by the activation of Akt and MAPK [10,13,39,40]. Therefore, we examined whether Tat-BLVRA inhibits Akt and MAPK activation. Akt and MAPK activation was increased by H_2_O_2_; however, Tat-BLVRA significantly reduced Akt and MAPK activation (Figure 5).

### 2.5. Effects of Tat-BLVRA on Ischemic Insults

**Figure 6:**
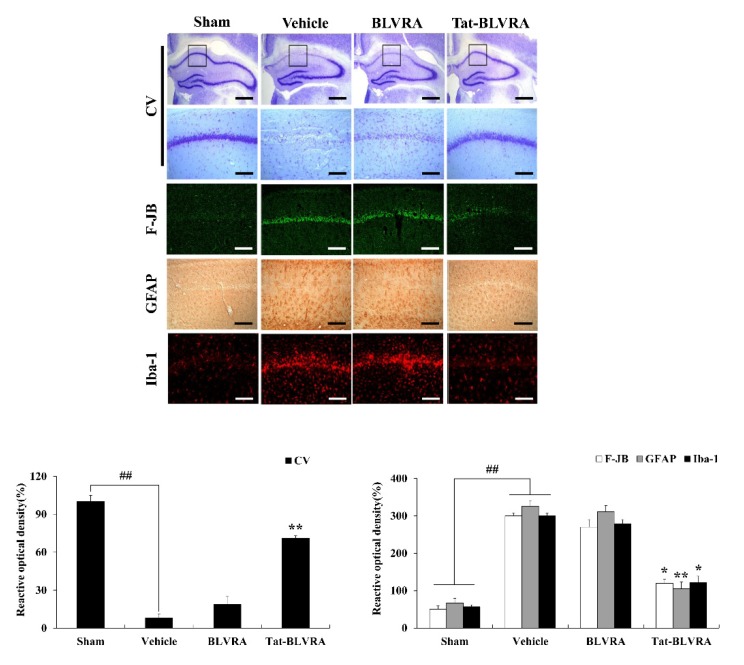
Effects of Tat-BLVRA protein on neuronal cell death in an animal model of ischemia. Gerbils were treated with a single injection of Tat-BLVRA and control BLVRA protein (2 mg/kg) before ischemia-reperfusion and sacrificed after 7 days. Neuronal cell viability was analyzed by cresyl violet (CV), fluoro-Jade B (F-JB), ionized calcium-binding adaptor molecule 1 (Iba-1), and glial fibrillary acidic protein (GFAP) immunostaining. Relative numeric analysis of CV-, F-JB-, Iba-1-, GFAP-positive neurons in the CA1 region is shown. Scale bar = 18.8 μm and 50 μm. ** *p* < 0.01 significantly different from the vehicle group. ^##^ *p* < 0.01 significantly different from the sham group.

The protective effect of Tat-BLVRA on ischemic injury was obtained by Cresyl violet (CV) and Fluoro-Jade B (F-JB) staining, which are known to be sensitive markers for neuronal damage [41,42]. Figure 6 shows that the Tat-BLVRA-treated group showed significantly increased CV-positive stained cells, whereas F-JB-positive stained cells showed the opposite pattern in the hippocampal CA1 region.

Further evidence to show the protection of Tat-BLVRA against ischemic injury, astrocytes, and microglia activation were measured. It is known that the activation of astrocytes and microglia are used as markers for the detection of ischemic injury [43,44]. The Tat-BLVRA-treated group demonstrated drastically decreased ionized calcium-binding adaptor molecule 1 (Iba-1) and glial fibrillary acidic protein (GFAP)-positive stained cells. The control BLVRA-treated group showed no change compared to the vehicle group.

## 3. Discussion

Biliverdin reductase converts biliverdin to bilirubin and has two isozymes, biliverdin reductase A (BLVRA) and biliverdin reductase B (BLVRB), which are abundantly expressed in various tissues [2,3]. Biliverdin reductase is involved in the processing of various diseases [13,14,15,19,20,21] and other groups have reported that overexpression of biliverdin reductase has a protective function in hypoxia by regulation of apoptosis via extracellular signal-regulated kinase (ERK) signal pathways [45]. In addition, biliverdin reductase is associated with metabolic diseases by its connection with a wide range of cellular singling pathways, including insulin receptor kinase cascades, protein kinase cascade, and inflammatory mediators [3]. Recently, biliverdin reductase showed that this enzyme has an antioxidant role in hippocampal neuron survival in Alzheimer disease (AD). However, impairment of biliverdin reductase is a common clinical feature in the symptomatology of AD and type 2 diabetes mellitus (T2DM). These reports suggest that biliverdin reductase is important in the prevention of AD and T2DM [8,9,13]. Even though biliverdin reductase is involved in various diseases, the role of this enzyme in ischemic insults has not been investigated yet. Many reports showed that various PTD-fused target proteins can be transduced into cells [22,25,26,27,28,29,30,31,32,33,34,35,36]. Thus, we determined whether cell permeable protein transduction domain (PTD) Tat fused with BLVRA (Tat-BLVRA) has a protective effect against hippocampal neuronal cell death.

We showed that Tat-BLVRA was efficiently transduced into HT-22 cells. Also, we confirmed that transduced protein was distributed in both the nucleus and cytosol. Oxidative stress induces ROS generation, and elevation of ROS finally leads to cell death. Excessive elevation of ROS is a major risk factor in various diseases. Therefore, inhibition of ROS generation is an important strategy for cell survival [16,17,18,23,24]. In this study, we examined whether Tat-BLVRA inhibits H_2_O_2_-induced cell death. We showed that Tat-BLVRA markedly enhanced cell survival by inhibition of ROS production and DNA fragmentation. Other studies have reported that transfected biliverdin reductase protected against oxidative stress-induced HeLa cell death, whereas cell death was increased by significant elevation of ROS production when the complementary RNA interference (RNAi) of biliverdin reductase was transfected into HeLa cells [1]. Also, another group has shown that biliverdin reductase increased pulmonary arterial smooth muscle cell (PASMC) survival under hypoxia by inhibition of DNA fragmentation in a biliverdin reductase-dependent manner [45]. Therefore, the results we obtained suggest that Tat-BLVRA protected against cell death via its antioxidant function.

Oxidative stress induces apoptotic responses leading to mitochondrial dysfunction and cell death [46] and it is well known that protein expressions of Bcl-2, Bax, and Caspase cascade are involved in apoptotic signaling pathways [37,38]. Thus, we investigated whether Tat-BLVRA recovered anti- and pro-apoptosis by up- or down-regulation of those proteins in H_2_O_2_-exposed HT-22 cells. Tat-BLVRA significantly increased Bcl-2 expression in H_2_O_2_-exposed cells, whereas Bax expression declined under the same conditions. In addition, Tat-BLVRA increased Caspase-8, -9, and -3 expression levels in a dose-dependent manner in H_2_O_2_-exposed cells. It has been reported that biliverdin reductase contributes to the protective process against hypoxia on pulmonary arterial smooth muscle cells (PASMC) death via regulation of apoptosis signaling pathways, and biliverdin reductase promotes cell survival by inhibiting the activation of Caspase-3 [45,47].

Several studies have demonstrated that biliverdin reductase is involved in MAPK and Akt signaling pathways [19,20,39], and we also showed that Tat-BLVRA regulated Akt and MAPK signaling pathways. These signaling pathways are known to be involved in oxidative stress, cell death, and cancer cell proliferation, suggesting that biliverdin reductase offers a novel target molecule for the inhibition of cancer cell growth [6,48,49,50]. Even though many studies have suggested the connection of biliverdin reductase to signaling pathways, the protective mechanism of BLVRA in cell survival remains to be elucidated.

We have already demonstrated that various PTD-fused proteins protected against neuronal cell death in ischemic animal models [31,33,34]. Barone et al. have reported that oxidative stress-induced impairment of BLVRA in the hippocampus and decreased BLVRA would have deleterious effects in AD, suggesting that BLVRA is an effective therapeutic strategy proposing to improve AD pathology as a powerful antioxidant [11]. Other studies have shown that BLVRA ameliorates the pathological signs in the progression of AD by reduction of ROS, whereas dysfunction or loss of BLVRA results in a loss of neuroprotection in AD by increased ROS [9,11]. Also, overexpression of BLVRA has similar protective effects in fibroblast cells by oxidative stress [5]. However, the protective effect of BLVRA on other neuronal damage induced by ischemic injury has not been studied yet. In this study, transduced Tat-BLVRA markedly protected cell death and inhibited activation of astrocytes and microglia in the hippocampal CA1 region of an ischemic animal model. Other studies have reported that astrocyte and microglia activation occur in the hippocampus CA1 region during ischemic insults [51,52].

Based on our results, Tat-BLVRA protected hippocampal neuronal cell death from oxidative stress, suggesting that BLVRA may provide a novel therapeutic agent for ischemia.

## 4. Materials and Methods

### 4.1. Cell Culture and Viability Measurements

Hippocampal neuronal HT-22 cells (Korean Cell Line Bank, Seoul, Korea) were cultured in Dulbecco’s modified Eagle’s medium (DMEM) with 5 mM NaHCO_3_, 20 mM N-2-hydroxyethil-piperazine-N’-2-ethanesulfonic acid (HEPES)/NaOH (pH 7.4), 10% fetal bovine serum (FBS), and antibiotics. After treatment of Tat-BLVRA (1–5 µM) and control BLVRA (1–5 µM), cell viability was estimated by 3-(4,5-dimethylthiazol-2-yl)-2,5-diphenyl terazolium bromide (MTT) assay [31].

### 4.2. Transduction of Tat-BLVRA into HT-22 Cells

Tat-BLVRA and control BLVRA was purified as described previously [19]. Transduction of Tat-BLVRA was observed in HT-22 cells after pretreatment of fusion protein and transduced protein was confirmed as described previously [31].

### 4.3. Western Blot Analysis

The proteins were resolved by sodium dodecyl sulfate-polyacrylamide-gel electrophoresis (SDS-PAGE), transferred onto nitrocellulose membrane, and subsequently incubated with primary antibodies: His (1:5,000; sc-804; Santa Cruz Biotechnology), Akt (1:2,000; #9273), p-Akt (1:2,000; #4058), JNK (1:1,000; #9258), p-JNK (1:1,000; #9251), ERK (1:2,000; #9102), p-ERK (1:2,000; #4376), p38 (1:2,000; #9212), p-p38 (1:2,000; #4631), Bcl-2 (1:1,000; #2876), Bax (1:1,000; #2772), Caspase-3 (1:1,000; #9662), Caspase-8 (1:1,000; #4927S), Caspase-9 (1:1,000; #9504S), β-actin (1:5,000; #4967), and appropriate secondary antibodies (1:10,000; #7074). All of the above antibodies were purchased from the Cell Signaling Technology (Beverly, MA, USA), except for the His antibody. Protein bands were detected by the method described in a previous study [31,52].

### 4.4. Measurement of Reactive Oxygen Species (ROS) and DNA Fragmentation

Intracellular ROS level and DNA fragmentation were measured using 2′,7′-dichlorodihydrofluorescein diacetate (DCF-DA) and terminal deoxynucleotidyl transferase mediated dUTP nick end labeling (TUNEL) staining. HT-22 cells (1 × 10^5^) were pretreated with Tat-BLVRA (5 µM) and control BLVRA (5 µM) for 2 h and exposed to hydrogen peroxide (1 mM). Then, DCF-DA and TUNEL staining was performed as described previously [31,34].

### 4.5. Measurement of Activation of Akt and MAPK as well as Apoptosis Signals

The expression levels of Akt (10 min), c-Jun N-terminal kinase (JNK) (30 min), ERK (30 min), p38 (10 min), Bcl-2 (1 h), Bax (1 h), caspase-3 (10 min), caspase-8 (20 min), and caspase-9 (1 h) in Tat-BLVRA- (1–5 µM) and control BLVRA (1–5 µM)-treated cells were analyzed using the indicated antibodies. Using a densitometer (Image Lab version 5.2, Bio-Rad Laboratories, Hercules, CA, USA), the bands were measured.

### 4.6. Experimental Animals

Male gerbils (65–75 g) used in this experiment were cared for and approved by the Institutional Animal Care and Use Committee of Soonchunhyang University (SCH 15-0006). To examine whether Tat-BLVRA protects against ischemic insults, gerbils were divided into four groups (*n* = 7 per group): sham-, vehicle-, Tat-BLVRA-, and control BLVRA-treated groups. Tat-BLVRA (2 mg/kg) or control BLVRA (2 mg/kg) was intraperitoneally injected before ischemia-reperfusion, as described previously [31,34].

### 4.7. Statistical Analysis

The measurement of immunoreactive cells was conducted as described previously [27,30]. Data are expressed as the mean ± standard error of the mean (SEM) of three different experiments. The data were analyzed using one-way analysis of variance (ANOVA) and student’s t-test to determine statistical significance. Bonferroni’s test was used for post-hoc comparisons (GraphPad Prism 8; GraphPad Software Inc., La Jolla, CA, USA). * *p* < 0.05 or ** *p* < 0.01 was considered to indicate a statistically significant difference.
